# Syndecan-1 is up-regulated in ras-transformed intestinal epithelial cells.

**DOI:** 10.1038/bjc.1998.147

**Published:** 1998-03

**Authors:** Z. M. Wong, B. Choo, M. Li, D. J. Carey, D. F. Cano-Gauci, R. N. Buick

**Affiliations:** Ontario Cancer Institute and Department of Medical Biophysics, University of Toronto, Canada.

## Abstract

**Images:**


British Journal of Cancer (1998) 77(6), 890–896
© 1998 Cancer Research Campaign

### Syndecan-1 is up-regulated in *ras*-transformed intestinal epithelial cells

ZM Wong[1], B Choo[1], M Li[1], DJ Carey[2], DF Cano-Gauci[1] and RN Buick[1]

[1]Ontario Cancer Institute and Department of Medical Biophysics, University of Toronto, 610 University Ave., Toronto, ON, M5G 2M9; [2]Sigfried and Janet Weis Centre for Research, Geisinger Clinic, Danville, PA 17822, USA

**Summary** The syndecans, a family of cell-surface heparan sulphate proteoglycans, have been proposed to mediate cellular interactions with extracellular effector molecules, such as growth factors and components of the extracellular matrix, during critical phases of development. Transcripts of all four syndecans are expressed at varying levels in the developing rat intestine and in a series of immature rat intestinal epithelial cell lines. In addition, we report the novel finding that, in the intestinal epithelial cell lines, expression of syndecan-1 transcript is up-regulated by transformation with activated H-*ras*. This is in contrast to other cell lines in which *ras* transformation is associated with a decrease in syndecan-1 levels. The observed increase in the syndecan-1 occurs as a result of increased transcription and can be correlated with the degree of transformation of the IEC-18 cells. Transformation is also associated with a decrease in apparent molecular weight and increased shedding of the proteoglycan into the culture medium. Increased shedding of syndecan-1 into the culture medium after transformation with H-*ras* may contribute to the disruption of proteoglycan interactions with the extracellular matrix, leading to alterations in cell adhesion and organization.

**Keywords:** proteoglycan; syndecan; *ras* transformation; intestinal epithelial cell

In the rat intestine, dramatic changes over the last few days of gestation result in the reorganization of a tubular structure into the mature crypt-villus structure in which the mesenchyme is lined by a simple columnar epithelium (Moog, 1979; Trier and Moxey, 1979; Madara et al, 1981). Underlying this morphogenesis and the maintenance of the adult crypt-villus structure are reciprocal interactions between the epithelium and mesenchyme (Kedinger et al, 1986) that involve recognition phenomena between cell-surface molecules and components of the pericellular environment. Membrane-bound proteoglycans contribute to the regulation of cell behaviour through interactions with extracellular matrix components or by acting as co-receptors for biologically active peptides (Klagsbrun and Baird, 1991; Rapraeger et al, 1991; Yayon et al, 1991; Aviezer et al, 1994).

Two gene families encoding cell-surface heparan sulphate proteoglycans (HSPG) have been identified on the basis of sequence similarities between their core proteins: the glypican-related integral membrane proteoglycans (PGs) and syndecan-like integral membrane PGs (David,1993). We have reported that OCI-5, a glypican-related HSPG (Filmus et al, 1995), is involved in intestinal development (Filmus et al, 1988). Although syndecans-1 and -4 have been detected in the adult intestine (Kim et al, 1994), the involvement of the syndecan family in intestinal development or differentiation is largely unknown. The role of syndecan-1 in epithelial–mesenchymal interactions during morphogenesis of a variety of tissues raises the possibility of a similar role in the developing intestine. The recent cloning of the four members of the syndecan gene family (Carey et al, 1992; Kojima et al, 1992; Pierce et al, 1992) makes it feasible to analyse the expression of this family in the intestine and in intestinal models. This work describes the expression of syndecans in intestinal systems and the effect of *ras* transformation on the expression of syndecan-1 in intestinal epithelial cells (IECs).

### MATERIALS AND METHODS

### Materials

Rat intestinal epithelial cell (IEC) lines IEC-6, -14, -17, -18, -19 and -20 (Quaroni et al, 1979; Quaroni and Isselbacher, 1981) were obtained from Dr A Quaroni (Cornell University); Rat2 fibroblasts, FR, fetal rat skin fibroblasts and NRK-52E from ATCC. Rat syndecan-1 and syndecan-4 cDNA (Kojima et al, 1992) were obtained from Dr Rosenberg (MIT) and rat syndecan-2 cDNA (Pierce et al, 1992) was obtained from Dr Cowling (Christie Hospital, Manchester, UK). Rat syndecan-3 cDNA (Carey et al, 1992) has been described. Sprague–Dawley rats were purchased from Charles River. Enzymes were from ICN Chemicals. Serum and Trizol reagent were from Gibco/BRL. Radioisotopes and Renaissance chemiluminescence reagents were from NEN. All other reagents were from Sigma Aldrich.

### Cell culture

Cell lines were cultured in a humidified chamber at 37°C in 5% carbon dioxide. IEC lines were grown in alpha minimal essential medium (α-MEM) supplemented with 3.6 mg $ml^{-1}$ glucose, 0.3 mg/$ml^{-1}$ glutamine, 0.27 U $ml^{-1}$ Humulin (Eli Lilly) and 5% fetal bovine serum (Quaroni et al, 1979). Culture conditions for *ras*-transformed IEC-18 cells have been described (Buick et al, 1987). FR, Rat2 and NRK-52E cells were grown in α-MEM supplemented with 10% fetal bovine serum.

Received 24 March 1997
Revised 26 August 1997
Accept 28 August 1997

Correspondence to: ZM Wong, 178 Glengrove Avenue, Toronto, Ontario, Canada M4R 1P3

In memory of Dr Ronald N Buick, 12 March, 1948–20 July, 1996.

**Figure 1** Expression of syndecan-1, -2, -3 and -4 during intestinal development. Northern blot analysis of syndecan-1, -2, -3 and -4 in 17- and 20-day embryos, 8-day suckling and 24-day post-natal rat intestine. Tubulin probing was carried out to demonstrate equal loading

**Figure 2** Expression pattern of syndecans in IEC cell lines. Northern blot analysis of syndecan-1, -2, -3 and -4 in IEC-6, -14, -17, -18, -19 and -20 cells. Tubulin probing was carried out to demonstrate equal loading

### Northern blot analysis

RNA was prepared as described (Chomczynski and Sacchi, 1987) from approximately $10^7$ cells of each cell line or from samples of the whole developing rat intestine. Poly-A$^+$ RNA was prepared by adsorption on cellulose–oligo-dT columns. Then, 3 μg of polyA$^+$ RNA or 20 μg total RNA were separated by electrophoresis in 0.75% agarose–formaldehyde gels in phosphate buffer. The gel was blotted onto Zeta-probe (Bio-Rad) and prehybridized (45% formamide, 5 × Denhardt's, 2% sodium dodecyl sulphate (SDS), 5 × SSPE (Maniatis et al, 1989), and 100 μg/ml$^{-1}$ each of salmon sperm DNA and polyA$^+$ RNA) at 42°C for at least 1 h. For hybridization, $5 \times 10^5$ c.p.m. ml$^{-1}$ $^{32}$P-labelled syndecan or tubulin probe was boiled before addition to the buffer for a 16-h incubation at 42°C. Non-specifically bound probe was removed by washing for 30 min in 2 × standard saline citrate (SSC), 0.2% SDS at 42°C, 30 min in 2 × SSC, 0.2% SDS at 65°C and 30 min in 1 × SSC, 0.1% SDS at 65°C before exposing to Kodak X-Omat film at –70°C with intensifying screens. Relative molecular mass was determined relative to the 18S and 28S ribosomal RNA.

### Probes

The 585-bp *Kpn*l–*Eco*R1 fragment of pNWS127 was used as the syndecan-1 probe (Kojima et al, 1992). It hybridizes to a major band at 2.6 kb and a minor band at 3.4 kb, which may be obscured when syndecan-1 levels are high. The syndecan-2, probe which recognizes mRNA of 3.4, 2.2 and 1.1 kb, was the 2-kb *Eco*R1 fragment of the pBS plasmid into which it was cloned (Pierce et al, 1992); syndecan-3 probe was the 2-kb *Eco*R1 fragment from p104 and hybridizes to a 5.6-kb message (Carey et al, 1992); syndecan-4 probe, which hybridizes to a 2.6-kb mRNA, was the 228-bp *Xba*I-*Hin*dIII fragment from pNWS126 (Kojima et al, 1992). The H-*ras* probe was the *Bam*HI fragment of the c-H-*ras* oncogene cloned from EJ bladder carcinoma cell line (Shih and Weinberg, 1982). Either tubulin (Elliott et al, 1985) or GAPDH (Fort et al, 1985) were used as control probes. For analysis of the transcription rate, PUC18 was used as the negative control, while the 1.6-kb *Sac*1 fragment of L32 (Dudov and Perry, 1984) was used as the positive standard. The DNA fragments were labelled with [$^{32}$P]-dCTP by random priming (Feinberg and Vogelstein, 1983; 1984). Nuclear run-ons were carried out as described (Hu et al, 1995).

### Preparation of PG-enriched extracts from conditioned medium and cultured cells

Conditioned medium was harvested from confluent cultures of IEC-18 and its *ras*-transformed clones. After debris was removed by low-speed centrifugation, the medium was supplemented with Tris-buffered saline (TBS), 20 mM EDTA at pH 8, 5 mM phenylmethyl-sulphonyl fluoride (PMSF) and mixed with DEAE-sephacel (5-ml beads per liter of medium) overnight at 4°C with gentle stirring. The beads were recovered by low speed centrifugation, washed with TBS plus 0.4 M sodium chloride and protease inhibitors (20 mM EDTA, pH 8, 0.2 mM PMSF, 10 mM *N*-ethylmaleimide, 5 mM benzamidine hydrochloride and 5 mM 6-aminohexanoic acid) and

© Cancer Research Campaign 1998

eluted with the same buffer containing 2 M sodium chloride. The eluate was then concentrated on an Amicon Centriprep-50 at 4°C. For cell extracts, confluent cells were harvested by scraping into TBS, rinsed with TBS and $10^7$ cells extracted in 100 µl of 1% Triton-X 100 in 50 mM Tris, pH 7.4, and protease inhibitors (as above) at 4°C for 30 min. Insoluble material was pelleted in a microcentrifuge for 15 min at 14 000 r.p.m. and the supernatant boiled for 10 min in SDS-PAGE loading buffer before gel electrophoresis. Each sample represents material extracted from $5 \times 10^6$ cells.

### Enzyme digestion of PG extracts

PG-enriched extracts were exchanged into TBS supplemented with protease inhibitors, except EDTA. To each 50-µl extract was added 10 mU of chondroitinase ABC for 1 h at 37°C. For digestion with heparitinase, 20 mM calcium acetate and 1 mU of heparitinase were added for 1 h at 40°C.

### Western blot analysis of PG extracts

PGs electrophoretically separated on polyacrylamide gels were transferred overnight onto Immobilon (Millipore, Mississauga, ON, Canada) in half-strength Towbin buffer (Towbin et al, 1979) at 250 mA at 4°C. The blots were blocked with 3% Carnation non-fat milk, 0.05% Tween-20, in PBS for 30–60 min before incubating with antibodies diluted in the same solution for 2 h at room temperature or overnight at 4°C. After three washes with 0.05% Tween-20 in PBS, the blots were incubated for 30–45 min with a 1:1000 dilution of horseradish–peroxidase-labelled goat anti-rabbit secondary antibodies in blocking solution. The blots were washed three to five times with large volumes of 0.05% Tween-20 in PBS and incubated with Renaissance enhanced chemiluminescence solutions before exposure to radiographic film.

### RESULTS

### Transcript levels in developing rat intestine and intestinal epithelial cells

To investigate the potential role of the syndecan gene family in intestinal cell behaviour, we first analysed the levels of syndecan transcripts in whole fetal and adult rat intestine. Northern blot analysis (Figure 1) shows that transcripts of all four syndecans are expressed in the intestine but the levels of syndecans-2, -3 and-4 are higher during fetal development and through the suckling period than in the mature intestine (24 days post natal). Syndecans-2, -3 and-4 were barely detectable in the adult intestine, while syndecan-1 was expressed in both fetal and adult intestine.

We then analysed the levels of syndecan transcripts in IEC, a series of immortalized rat intestinal epithelial cells that have been widely used as models of developing intestinal crypt cells. Northern blot analysis (Figure 2) shows that transcripts of all four syndecans were widely expressed but at varying levels in the six IEC lines. The sizes of individual transcripts detected were consistent with those previously published. The 2.6-kb syndecan-1 transcript was present at roughly equivalent levels in all cell lines. Smaller amounts of the minor 3.4-kb transcript were also observed. The 3.4-, 2.2-, and 1.1-kb syndecan-2 transcripts showed a wide range of expression, being detected at very high levels in IEC-14, at slightly lower levels in IEC-18, -19, and -20, and at low levels in IEC-6 and -17. In contrast, the 5.6-kb syndecan-3 transcript was expressed at high levels in only IEC-6 cells. High levels of the 2.6-kb syndecan-4 transcript were detected in IEC-6, -18 and -19 with moderate levels present in IEC-14, -17 and -20. Each of the cell lines had a distinct complement of transcripts of the four syndecans. As with expression in the intestine, there was no obvious pattern associating high levels of one syndecan with another or mutual exclusion of any of the other syndecan transcripts.

**Figure 3** Expression of syndecan-1 transcripts in IEC-18 cells before and after transformation with activated H-*ras*. Northern blot analysis of syndecan-1 expression in *ras*-transformed IEC-18 clones arranged in increasing order of *ras* and corresponding increase in malignant phenotype. The Northern blot was also probed with GAPDH to demonstrate equal loading and with H-*ras* to facilitate correlation of levels of expression of H-*ras* and syndecan-1 transcripts

### Effect of malignant transformation on transcript levels of syndecan-1

Our laboratory has developed a model of malignant transformation of intestinal cells based on the expression of activated human H-*ras* in IECs (Buick et al, 1987). These transformed cells show altered expression of several cell-surface molecules involved in cell–cell or cell–matrix interactions. These include the down-regulation of OCI-5 (Filmus et al, 1988), a member of the glypican family of integral membrane PGs (David, 1993), and up-regulation of CD44 (Jamal et al, 1994). Syndecan-1 has been reported to function as an adhesion receptor for extracellular matrix in epithelial cells. Syndecan-1 transcripts were expressed by all the IEC lines examined. We further investigated the effect of malignant transformation on syndecan-1 expression using three *ras*-transformed cell lines derived from IEC-18, i.e. IEC-*ras*-3, IEC-*ras*-4 and IEC-*ras*-7. The relative levels of *ras* protein expression in these cell lines correspond to the degree of malignant transformation (Buick et al, 1987). The IECs changed from cuboidal to fusiform morphology after *ras* transformation and produced rapidly growing tumours in syngeneic rats or nude mice. As shown in Figure 3, the level of syndecan-1 transcript accumulation

© Cancer Research Campaign 1998

**Figure 4** Northern blot analysis of dexamethasone induced *ras* transformation. Clone 25 (IEC-18 transfected with H-*ras* under the control of MMTV promoter) cells were induced with 1 μM dexamethasone for the times (h) indicated. GAPDH probing was carried out to demonstrate equal loading

**Figure 5** Regulation of syndecan-1 expression in IEC-18 cells before and after H-*ras* transformation. Nuclear run-ons were used to determine the effect of *ras* transformation on transcriptional rate. The ratio of signals in non-transformed compared with transformed was normalized to L32

increases in tandem with increasing levels of *ras* expression. Transcript levels of the other syndecans did not change as dramatically as syndecan-1 upon transformation of IEC-18 with *ras* (data not shown). The effect of *ras* expression on syndecan-1 transcript accumulation was also confirmed in a clone transfected with H-*ras* under the control of the MMTV promoter. Figure 4 shows that syndecan-1 transcript levels increased when *ras* expression was induced with dexamethasone for 12 h, an increase still evident after 48 h. This increase in syndecan-1 transcripts after *ras* transformation is a novel finding: previous reports have indicated that *ras* transformation of colonic (Levy et al, 1996) or mammary epithelial cells (Kirjavainen et al, 1993) results in decreased membrane-anchored syndecan-1 but unaltered transcript levels (see Discussion).

### Effect of *ras* transformation on transcriptional rate of syndecan-1

Syndecan-1 levels have been reported to be regulated at the transcriptional, post-transcriptional (Sanderson et al, 1992; Vainio et al, 1992; Yeaman and Rapraeger, 1993) and post-translational levels (Sanderson and Bernfield, 1988; Bernfield and Sanderson, 1990; Inki et al, 1992; Kirjavainen et al, 1993). Nuclear run-on experiments (Figure 5) show that *ras* transformation of IEC-18 cells resulted in a 9.6-fold increase in the transcription rate, similar to the increase in syndecan-1 transcripts shown by Northern blot analysis. Therefore, syndecan-1 is regulated at the transcriptional level in *ras*-transformed IEC-18 cells.

### Effect of malignant transformation on syndecan-1

The relationship of elevated syndecan-1 transcript in *ras*-transformed cells to PG expression was analysed using an antiserum specific for the syndecan-1 ectodomain (Carey et al, 1994). The Western blot in Figure 6 showed that the transcriptional up-regulation of syndecan-1 by transformation with *ras* was associated with an increase in the corresponding protein. The antiserum used recognized the intact syndecan-1 as a high $M_r$ smear characteristic of proteoglycans (Figure 6). In contrast, a sharper and lower $M_r$ band was seen after chondroitinase ABC and heparitinase digestion of syndecan-1 GAG chains (see Figure 7). While very little syndecan-1 was present in either the IEC-18-conditioned medium (Figure 6A) or cell lysate (Figure 6B), the level of syndecan-1 increased in the transformed cell lines. The distribution of syndecan-1 was also altered in relationship to levels of *ras* expression. In IEC-*ras*-3, the least transformed clone used (Buick et al, 1987), the increase in syndecan-1 expression was at the cell surface with little shed into the medium. In contrast, a greater proportion of syndecan-1 was shed into the growth medium, with little resident on the cell surface of the highly transformed IEC-*ras* 7. In IEC-*ras*-4 cells, which had intermediate *ras* levels, syndecan-1 of intermediate $M_r$ was evident both at the cell surface and in conditioned medium. Moreover, cells expressing higher levels of *ras* expressed syndecan-1 of a lower apparent $M_r$: the apparent $M_r$ of syndecan-1 was over 200 kDa for IEC-18 and IEC-*ras*-3, approximately 200 kDa for IEC-*ras*-4 and less than 200 kDa for IEC-*ras*-7. The immunoreactive species in the medium was smaller than that in the cell extracts, especially in IEC-*ras*-7 cells. An increased $M_r$ of syndecan-1 has previously been reported for both *ras*-transformed mouse keratinocytes (Inki et al, 1992) and mouse mammary (NOG) cells (Kirjavainen et al, 1993), accompanied by a decreased amount of PG.

### GAG chain substitution of syndecan-1

Syndecan-1 is generally expressed as a hybrid PG bearing both CS and HS chains (Rapraeger et al, 1985). To analyse the syndecan-1 GAG chain substitutions, conditioned medium from IEC-*ras*-7 cells was digested with heparitinase, chondroitinase ABC or a combination of both. The Western blot in Figure 7 shows that the $M_r$ of syndecan-1 isolated from the culture medium of IEC-*ras* 7 cells decreased slightly after digestion with heparitinase but decreased more when a combination of chondroitinase and heparitinase was used. As chondroitinase alone did not result in detectable reduction in size of the syndecan-1, HS digestion may be required to expose the CS chains to degradation. These data indicate that the core protein is substituted with both HS and CS as in other cells. Thus, the lower $M_r$ syndecan-1 expressed by the more highly *ras*-transformed IEC-18 cells does not appear to be a result of the loss of a specific type of GAG chain but may be due to an overall decrease in the length or number of chains.

© Cancer Research Campaign 1998

**Figure 6** Detection of syndecan-1 in transformed IEC-18 cell lysates and conditioned medium. Western blot analysis of syndecan-1 from the conditioned medium (**A**) and cell surface (**B**) of IEC-18 cells and the three *ras*-transformed IEC-18 clones using an antiserum specific for the syndecan-1 ectodomain

**Figure 7** Enzyme susceptibility of syndecan-1 shed into the medium by IEC-*ras*-7 cells. Conditioned medium from IEC-*ras*-7 cells was digested with chondroitinase ABC, heparitinase or both before Western blotting using the syndecan ectodomain-specific antibody

### DISCUSSION

Expression of the four members of the syndecan family is regulated in diverse tissues at critical periods of development or remodelling (Elenius et al, 1991; Bernfield et al, 1992; Kim et al, 1994). To assess the potential contribution of the syndecan family to the complex epithelial–mesenchymal interactions during morphogenesis of the intestine, we analysed the expression of syndecan gene family members in intestinal models. In the whole rat intestine, transcripts of all four syndecans were detected during development. While syndecan-1 transcripts were expressed both during development and in the mature intestine, syndecan-2 and syndecan-3 transcripts were expressed at significantly higher levels during a period of morphogenetic change in the intestine, spanning day 17 of embryogenesis to 8 days post natal, and were subsequently down-regulated to barely detectable levels in the adult intestine. Syndecan-4 transcripts were up-regulated in the 8-day suckling intestine and then down-regulated in the mature intestine. The pattern of expression of the gene family in intestinal cells was complex, most likely reflecting differences in regulation of their expression. Each of the IEC lines analysed had a unique pattern of syndecan transcript expression, indicative of differences in the state of cellular differentiation and interactions with other cells and the extracellular matrix. The expression of each syndecan was independent of the others, with no apparent association or mutual exclusion. This contrasts with the complementary expression of syndecan-1 and syndecan-3 transcripts in the developing rat central nervous system (Carey et al, 1992), and with the co-ordinated expression of syndecan-1 and syndecan-2 in the developing lung mesenchyme (Bernfield et al, 1992; David et al, 1992;

*© Cancer Research Campaign 1998*

Kim et al, 1994). While the function of the syndecans during morphogenesis in the intestine is unknown, their expression and differential regulation is compatible with the notion that different combinations of the molecules are required at different stages. Although basement membrane HSPGs of the developing intestine are produced exclusively by the epithelial cells (Simon-Assmann et al, 1989), the cellular origin of syndecans is not clear. Their expression in IEC lines is consistent with their expression by the epithelium but expression by the mesenchyme and plasma cells, concurrently or at different stages, cannot be excluded. Application of in situ approaches will resolve this issue. Several syndecans may be involved during morphogenesis of the intestine, with the possible exception of syndecan-3, whose expression remains constant during embryogenesis and between IEC lines. Our current investigation has focused on the expression and regulation of syndecan-1 in intestinal models.

We report the novel finding that transformation of IEC-18 cells with H-*ras* resulted in a dramatic increase in the transcript levels of syndecan-1. In the series of increasingly malignant IEC-*ras*-3,-4 and -7 cells, the up-regulation of syndecan-1 increased in tandem with the level of expression of *ras* transcripts. Similar up-regulation of syndecan-1 transcripts upon *ras* transformation was observed in all IEC lines analysed and in FR cells but not in Rat2 fibroblasts or NRK-52E. This contrasts with the reported down-regulation of syndecan-1 transcripts in the human colonic carcinoma cell line Caco-2 after transformation with *ras* or PyMT (Levy et al, 1996). However, it is difficult to compare these results directly because the cell lines used represent different physiological situations: IEC-18 are non-tumorigenic cells, derived from normal epithelium, while Caco-2 were already malignant before transformation with *ras*. In addition, these conflicting results may reflect the multiple levels of regulation of syndecan-1 expression, including transcriptional control, post-transcriptionally and translationally or post-translationally. We determined that increased syndecan-1 transcript levels in H-*ras* transformed IEC-18 cells result from an increased transcription rate. Concomitant changes in $M_r$ and shedding of the molecule from the cell surface shows that the molecule is regulated at multiple levels. In contrast, in both *ras*-transformed NOG (Kirjavainen et al, 1993), and Caco-2 cells (Levy et al, 1996), there is a net decrease in the amount of membrane-anchored syndecan-1 but no alteration in mRNA levels. The up-regulation of syndecan-1 transcripts upon *ras* transformation of intestinal epithelial (IEC) and skin fibroblast (FR) cells but not of embryonic fibroblasts (Rat2) or epithelial-like kidney cells (NRK-52E) provides further evidence that the level of regulation of syndecan-1 is cell and tissue type specific.

In addition to changes in PG levels, the $M_r$ of syndecan-1 is altered in various transformed cell lines. The decrease in size of syndecan-1 in *ras*-transformed IEC-18 cells is consistent with the smaller syndecan-1 ectodomain expressed by *ras*-transformed Caco-2 cells (Levy et al, 1996) but contrasts with the increased $M_r$ due to altered glycosylation in transformed keratinocytes (Inki et al, 1992). *Ras* transformation of IEC-18 cells also results in increased shedding of syndecan-1 from the cell surface, similar to the effect of transformation in keratinocytes (Inki et al, 1992). This may be related to the acquisition of a less flattened morphology (Buick et al, 1987), consistent with previous reports of shedding of the syndecan-1 ectodomain upon cell rounding (Jalkanen et al, 1987). GAG chain substitution is an important component in syndecan-1 function. Generally, syndecans bear both CS and HS GAG chains. While CS chains are thought to be involved in detachability of cells and thus tumour metastasis, HS chains are associated with increased adhesion to extracellular matrix (Culp et al, 1978). However, HS levels are elevated in certain mammary carcinoma (Mangakis et al, 1990) and in a highly metastatic human melanoma cell line (Timar et al, 1992). Although enzymatic digestion of syndecan-1 isolated from IEC-*ras*-7 cells showed substitution with both CS and HS chains, alterations in both the size and subcellular localization of the molecules may affect their interactions with other molecules in the cellular microenvironment. Further experiments to determine the changes in structure and activity of the GAG chains and expression of cellular endoglycosidases will detect the more subtle effects of *ras* transformation that may affect cell shape and adhesion, and contribute to the malignant phenotype. It is important to note that the behaviour of cell lines in vivo does not necessarily mirror in vitro tests that gauge the transformed phenotype. A recent study of syndecan-1-transfected 293T (human embryonic kidney epithelial) cells found that, although the transformed cells grew better in reduced serum and showed reduced motility, these cells were tumorigenic when injected into nude mice (Numa et al, 1995). *Ras*-transformed IEC-18 cells are tumorigenic in nude mice (Buick et al, 1987), while the parent cells are not.

In summary, we have analysed syndecan expression in intestinal cells and tissues. The syndecans were expressed at varying levels in IEC lines and in the developing intestine, leading us to speculate that they play specific roles at discrete developmental stages. We report the novel finding that *ras* transformation of IEC-18 cells resulted in up-regulation of syndecan-1 but not syndecans-2, -3 or -4. This appears to be the result of an increased transcriptional rate. In addition, syndecan-1 expressed by *ras* transformants showed decreased $M_r$ and increased shedding into the medium. The concurrent expression and differential regulation of syndecans in both normal and transformed phenotypes are indicative of a complexity that will have to be addressed in determining the role of any one component in differentiation and development of the intestine. Experiments aimed at understanding the functional significance of syndecan-1 expression in relation to *ras* transformation may provide further information on processes involved in malignant transformation.

### ABBREVIATIONS

CS, chondroitin sulphate; GAG, glycosaminoglycan; HS, heparan sulphate; HSPG, heparan sulphate proteoglycan; IEC, intestinal epithelial cell; PG, proteoglycan; PMSF, phenylmethylsulphonyl fluoride

### ACKNOWLEDGEMENTS

We thank Rose Pullano for expert technical assistance and Drs RD Rosenberg and GJ Cowling for their generous gifts of syndecan cDNAs. This work was supported by studentships to ZMW and ML, grant MA8162 to RNB from the Medical Research Council of Canada and a Terry Fox Program Project grant from the National Cancer Institute of Canada.

### REFERENCES

Aviezer D, Levy E, Safran M, Svahn C, Buddecke E, Schmidt A, David G, Vlodavsky I and Yayon A (1994) Differential structural requirements of heparin and heparan sulfate proteoglycans that promote binding of basic fibroblast growth factor to its receptor. *J Biol Chem* **269**: 114–121

© Cancer Research Campaign 1998

Bernfield M and Sanderson RD (1990) Syndecan, a developmentally regulated cell surface proteoglycan that binds extracellular matrix and growth factors. *Philos Trans R Soc Lond B Biol Sci* **327**: 171–186

Bernfield M, Kokenyesi R, Kato M, Hinkes MT, Spring J, Gallo RL and Lose EJ (1992) Biology of the syndecans: a family of transmembrane heparan sulfate proteoglycans. *Annu Rev Cell Biol* **8**: 365–393

Buick RN, Filmus J and Quaroni A (1987) Activated H-ras transforms rat intestinal epithelial cells with expression of alpha-TGF. *Exp Cell Res* **170**: 300–309

Carey DJ, Evans DM, Stahl RC, Asundi VK, Conner KJ, Garbes P and Cizmeci-Smith G (1992) Molecular cloning and characterization of N-syndecan, a novel transmembrane heparan sulfate proteoglycan. *J Cell Biol* **117**: 191–201

Carey DJ, Stahl RC, Cizmeci-Smith G and Asundi VK (1994) Syndecan-1 expressed in Schwann cells causes morphological transformation and cytoskeletal reorganization and associates with actin during cell spreading. *J Cell Biol* **124**: 161–170

Chomczynski P and Sacchi N (1987) Single-step method of RNA isolation by acid guanidinium thiocyanate–phenol–chloroform extraction. *Anal Biochem* **162**: 156–159

Culp LA, Rollins BJ, Buniel J and Hitri S (1978) Two functionally distinct pools of glycosaminoglycan in the substrate adhesion site of murine cells. *J Cell Biol* **79**: 788–801

David G (1993) Integral membrane heparan sulfate proteoglycans. *Faseb J* **7**: 1023–1030

David G, van der Schueren B, Marynen P, Cassiman JJ and van den Berghe H (1992) Molecular cloning of amphiglycan, a novel integral membrane heparan sulfate proteoglycan expressed by epithelial and fibroblastic cells. *J Cell Biol* **118**: 961–969

Dudov KP and Perry RP (1984) The gene family encoding the mouse ribosomal protein L32 contains a uniquely expressed intron-containing gene and an unmutated processed gene. *Cell* **37**: 457–468

Elenius K, Vainio S, Laato M, Salmivirta M, Thesleff I and Jalkanen M (1991) Induced expression of syndecan in healing wounds. *J Cell Biol* **114**: 585–595

Elliott EM, Sarangi F, Henderson G and Ling V (1985) Cloning of 11 alpha-tubulin gene sequences from the genome of Chinese hamster ovary cells. *Can J Biochem Cell Biol* **63**: 511–518

Feinberg AP and Vogelstein B (1983) A technique for radiolabeling DNA restriction endonuclease fragments to high specific activity. *Anal Biochem* **132**: 6–13

Feinberg AP and Vogelstein B (1984) 'A technique for radiolabeling DNA restriction endonuclease fragments to high specific activity'. Addendum. *Anal Biochem* **137**: 266–267

Filmus J, Church JG and Buick RN (1988) Isolation of a cDNA corresponding to a developmentally regulated transcript in rat intestine. *Mol Cell Biol* **8**: 4243–4249

Filmus J, Shi W, Wong ZM and Wong MJ (1995) Identification of a new membrane-bound heparan sulphate proteoglycan. *Biochem J* **311**: 561–565

Fort P, Marty L, Piechaczyk M, Sabrouty SE, Dani C, Jeanteur P and Blanchard JM (1985) Various adult tissues express only one major mRNA species from the glyceraldehyde-3-phosphate dehydrogenase multigenic family. *Nucl Acids Res* **13**: 1431–1442

Hu ZB, Yang GS, Li M, Miyamoto N, Minden MD and McCulloch EA (1995) Mechanism of cytosine arabinoside toxicity to the blast cells of acute myeloblastic leukemia: involvement of free radicals. *Leukemia* **9**: 789–798

Inki P, Gomez M, Quintanilla M, Cano A and Jalkanen M (1992) Expression of syndecan in transformed mouse keratinocytes. *Lab Invest* **67**: 225–233

Jalkanen M, Rapraeger A, Saunders S and Bernfield M (1987) Cell surface proteoglycan of mouse mammary epithelial cells is shed by cleavage of its matrix-binding ectodomain from its membrane-associated domain. *J Cell Biol* **105**: 3087–3096

Jamal HH, Cano-Gauci DF, Buick RN and Filmus J (1994) Activated ras and src induce CD44 overexpression in rat intestinal epithelial cells. *Oncogene* **9**: 417–423

Kedinger M, Simon-Assmann PM, Lacroix B, Marxer A, Hauri HP and Haffen K (1986) Fetal gut mesenchyme induces differentiation of cultured intestinal endodermal and crypt cells. *Dev Biol* **113**: 474–483

Kim CW, Goldberger OA, Gallo RL and Bernfield M (1994) Members of the syndecan family of heparan sulfate proteoglycans are expressed in distinct cell-, tissue-, and development-specific patterns. *Mol Biol Cell* **5**: 797–805

Kirjavainen J, Leppa S, Hynes NE and Jalkanen M, (1993) Translational suppression of syndecan-1 expression in Ha-ras transformed mouse mammary epithelial cells. *Mol Biol Cell* **4**: 849–858

Klagsbrun M and Baird A (1991) A dual receptor system is required for basic fibroblast growth factor activity. *Cell* **67**: 229–231

Kojima T, Leone CW, Marchildon GA, Marcum JA and Rosenberg RD (1992) Molecular cloning and expression of two distinct cDNA-encoding heparan sulphate proteoglycan core proteins from a rat endothelial cell line. *J Biol Chem* **267**: 4870–4877

Levy P, Munier A, Baron-Delage S, Di Gioia Y, Gespach C, Capeau J and Cherqui G (1996) Syndecan-1 alterations during the tumorigenic progression of human colonic Caco-2 cells induced by human Ha-ras or polyoma middle T oncogenes. *Br J Cancer* **74**: 423–431

Madara JL, Neutra MR and Trier JS (1981) Junctional complexes in fetal rat small intestine during morphogenesis. *Dev Biol* **86**: 170–178

Mangakis N, Sehrt B, Mangakis P, Bowe E and Kleemann I (1990) Determining the degree of malignancy of individual cases of mammary carcinoma on the basis of clinical, morphological and biochemical parameters. *Bulletin du Cancer* **77**: 235–242

Maniatis T, Fritsch EF and Sambrook J (1989) *Molecular Cloning: A laboratory manual*. Cold Spring Harbour Laboratory: Cold Spring Harbour, NY

Moog F (1979) The differentiation and redifferentiation of the intestinal epithelium and its brush border membrane. In *Development of Mammalian Absorptive Processes* vol 70, Elliot K and Whelan J (eds), pp.31–50. Excerptica Medica

Numa F, Hirabayashi K, Tsunaga N, Kato H, K, OR, Shao H, Stechmann-Lebakken C, Varani J, Rapraeger A and Dixit VM (1995) Elevated levels of syndecan-1 expression confer potent serum-dependent growth in human 293T cells. *Cancer Res* **55**: 4676–4680

Pierce A, Lyon M, Hampson IN, Cowling GJ and Gallagher JT (1992) Molecular cloning of the major cell surface heparan sulfate proteoglycan from rat liver. *J Biol Chem* **267**: 3894–3900

Quaroni A and Isselbacher KJ (1981) Cytotoxic effects and metabolism of benzo[a]pyrene and 7,12-dimethylbenz[a]anthracene in duodenal and ileal epithelial cell cultures. *J Natl Cancer Inst* **67**: 1353–1362

Quaroni A, Wands J, Trelstad RL and Isselbacher KJ (1979) Epithelioid cell cultures from rat small intestine Characterization by morphologic and immunologic criteria. *J Cell Biol* **80**: 248–265

Rapraeger A, Jalkanen M, Endo E, Koda J and Bernfield M (1985) The cell surface proteoglycan from mouse mammary epithelial cells bears chondroitin sulfate and heparan sulfate glycosaminoglycans. *J Biol Chem* **260**: 11046–11052

Rapraeger AC, Krufka A and Olwin BB (1991) Requirement of heparan sulfate for bFGF-mediated fibroblast growth and myoblast differentiation. *Science* **252**: 1705–1708

Sanderson RD and Bernfield M (1988) Molecular polymorphism of a cell surface proteoglycan: distinct structures on simple and stratified epithelia. *Proc Natl Acad Sci USA* **85**: 9562–9566

Sanderson RD, Hinkes MT and Bernfield M (1992) Syndecan-1, a cell-surface proteoglycan, changes in size and abundance when keratinocytes stratify. *J Invest Dermatol* **99**: 390–396

Shih C and Weinberg RA (1982) Isolation of a transforming sequence from a human bladder carcinoma cell line. *Cell* **29**: 161–169

Simon-Assmann P, Bouziges F, Vigny M and Kedinger M (1989) Origin and deposition of basement membrane heparan sulfate proteoglycan in the developing intestine. *J Cell Biol* **109**: 1837–1848

Timar J, Ladanyi A, Lapis K and Moczar M (1992) Differential expression of proteoglycans on the surface of human melanoma cells characterized by altered experimental metastatic potential. *Am J Pathol* **141**: 467–474

Towbin H, Staehelin T and Gordon J (1979) Electrophoretic transfer of proteins from polyacrylamide gels to nitrocellulose sheets: procedure and some applications. *Proc Natl Acad Sci USA* **76**: 4350–4354

Trier JS and Moxey PC (1979) Morphogenesis of the small intestine during fetal development. In *Development of Mammalian Absorptive Processes* vol 70, Elliot K and Whelan J (eds), pp.3–29. Excerptica Medica

Vainio S, Jalkanen M, Bernfield M and Saxen L (1992) Transient expression of syndecan in mesenchymal cell aggregates of the embryonic kidney. *Dev Biol* **152**: 221–232

Yayon A, Klagsbrun M, Esko JD, Leder P and Ornitz DM (1991) Cell surface, heparin-like molecules are required for binding of basic fibroblast growth factor to its high affinity receptor. *Cell* **64**: 841–848

Yeaman C and Rapraeger AC (1993) Post-transcriptional regulation of syndecan-1 expression by cAMP in peritoneal macrophages. *J Cell Biol* **122**: 941–950

© Cancer Research Campaign 1998


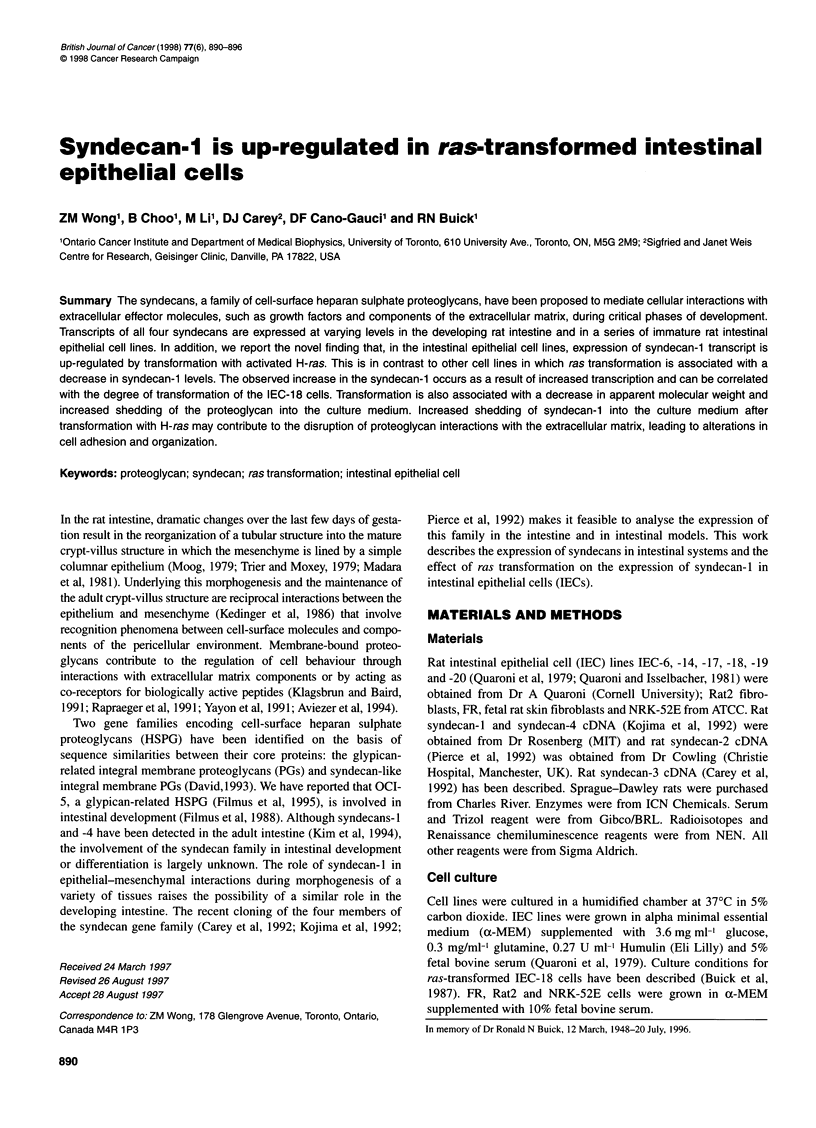

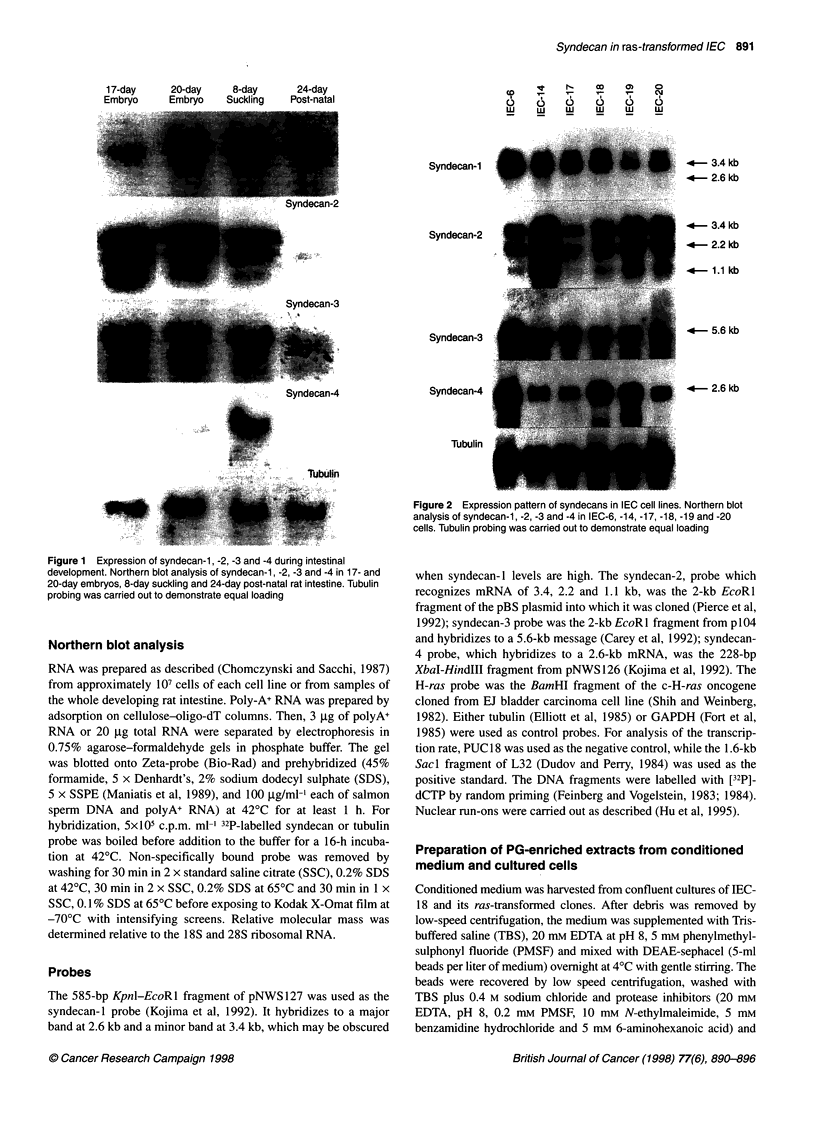

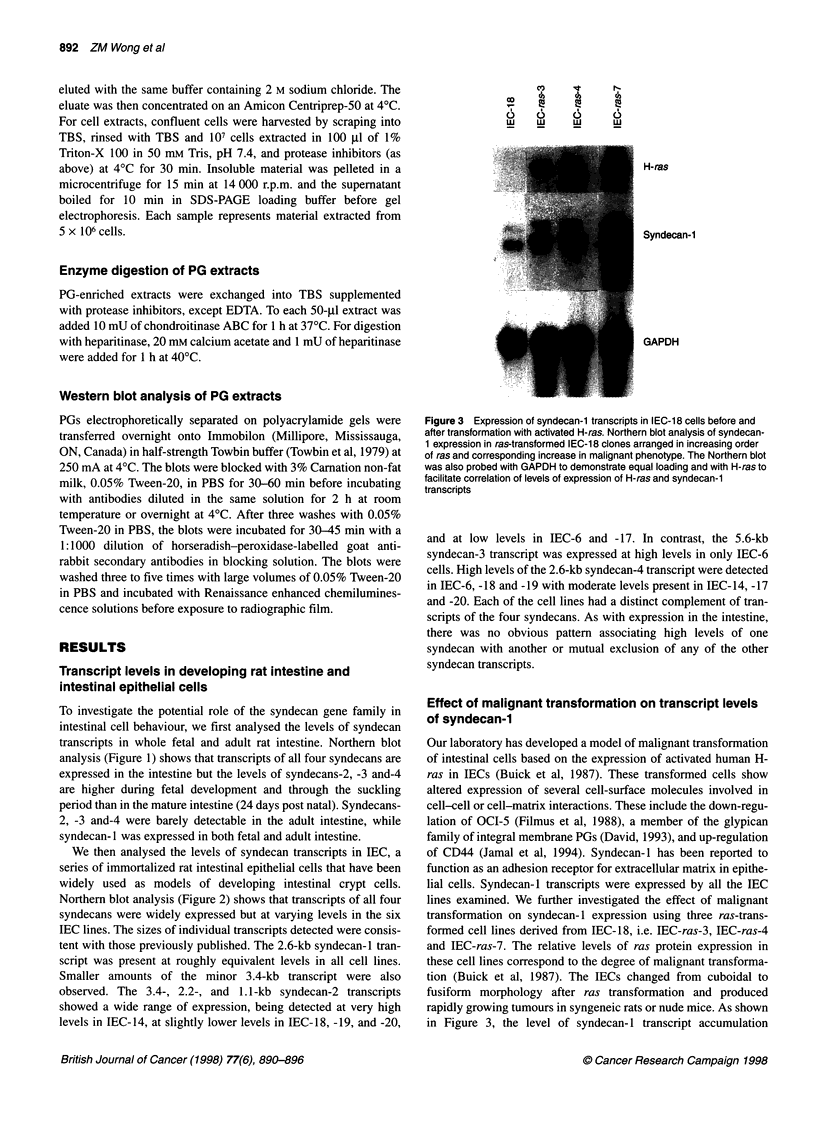

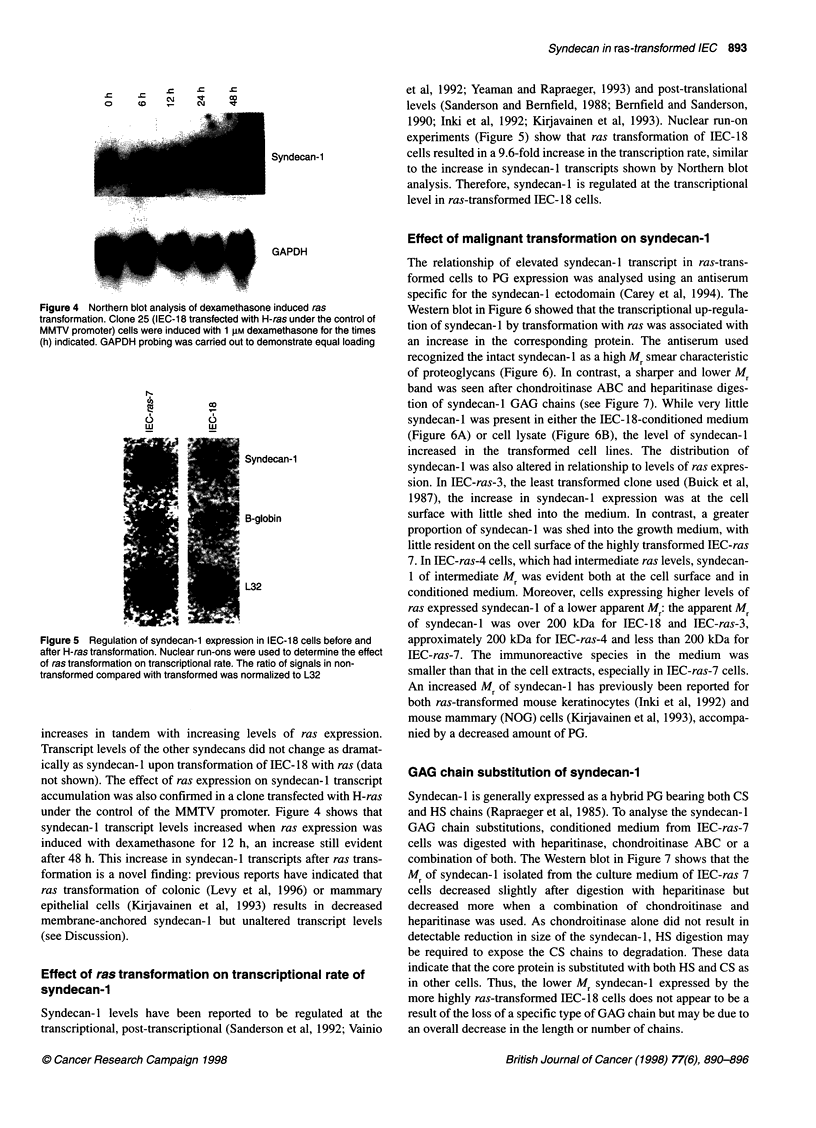

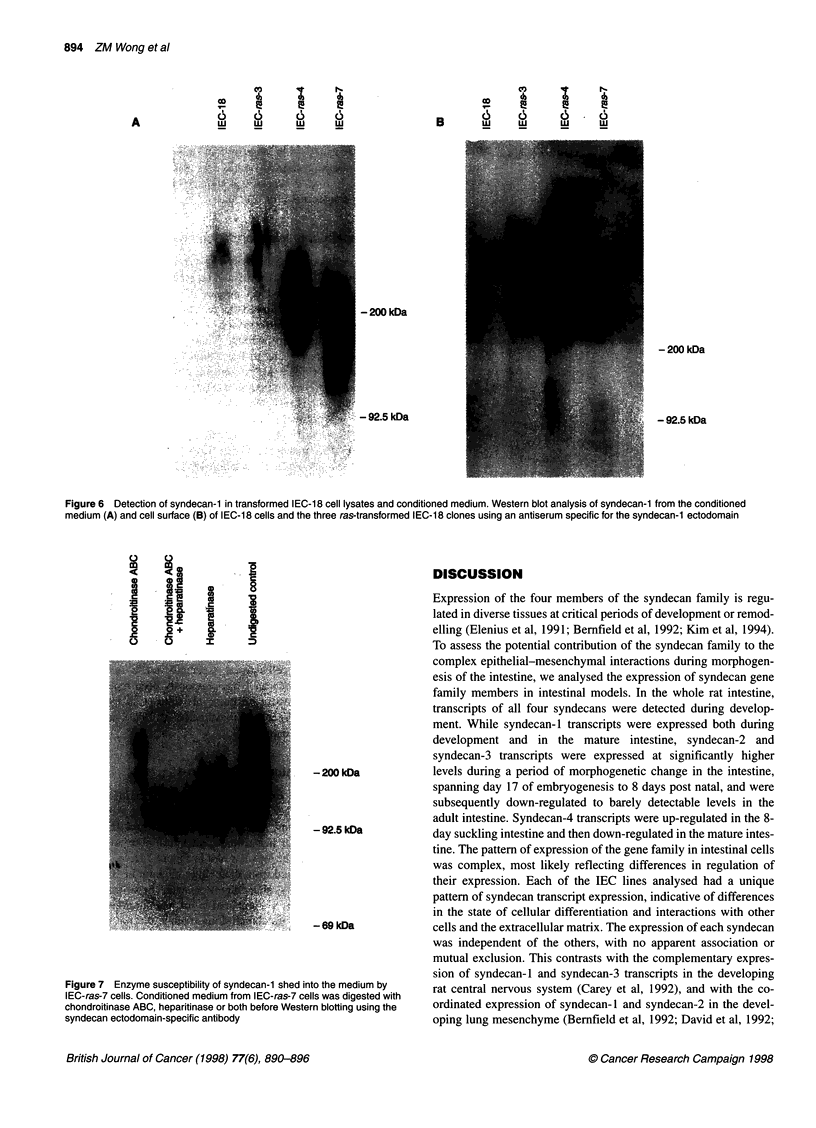

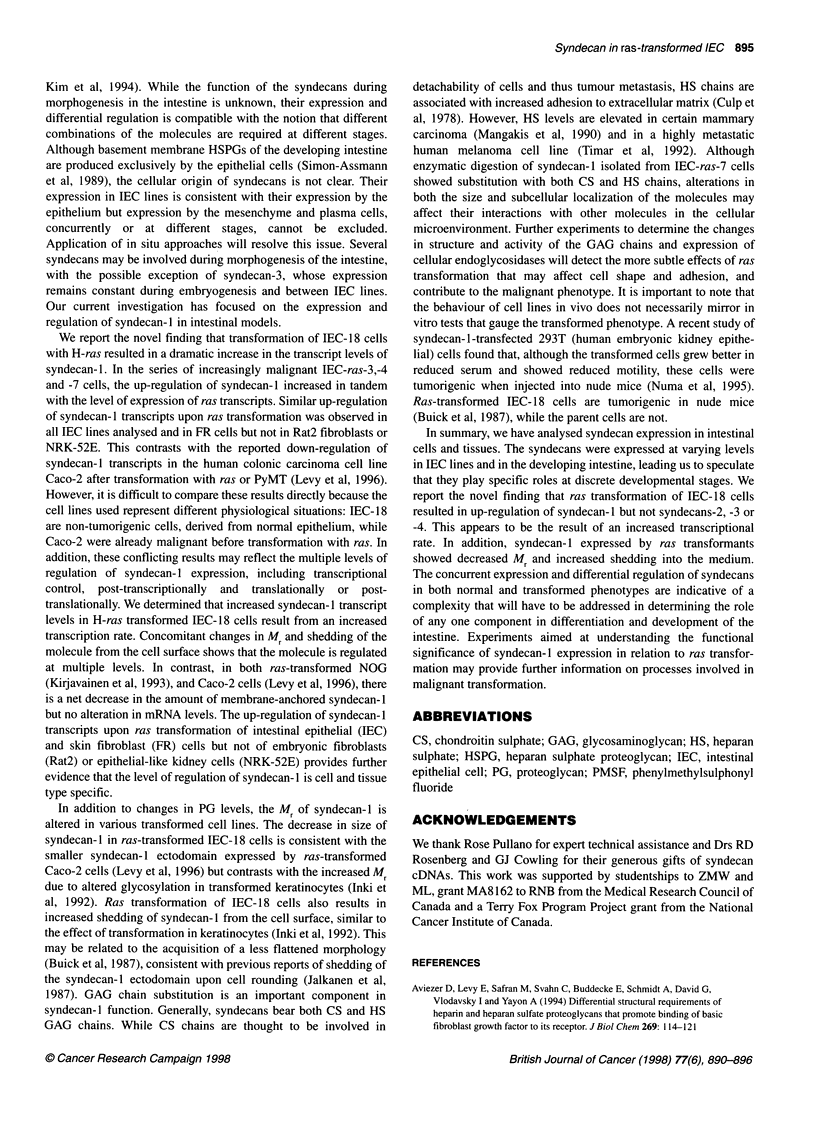

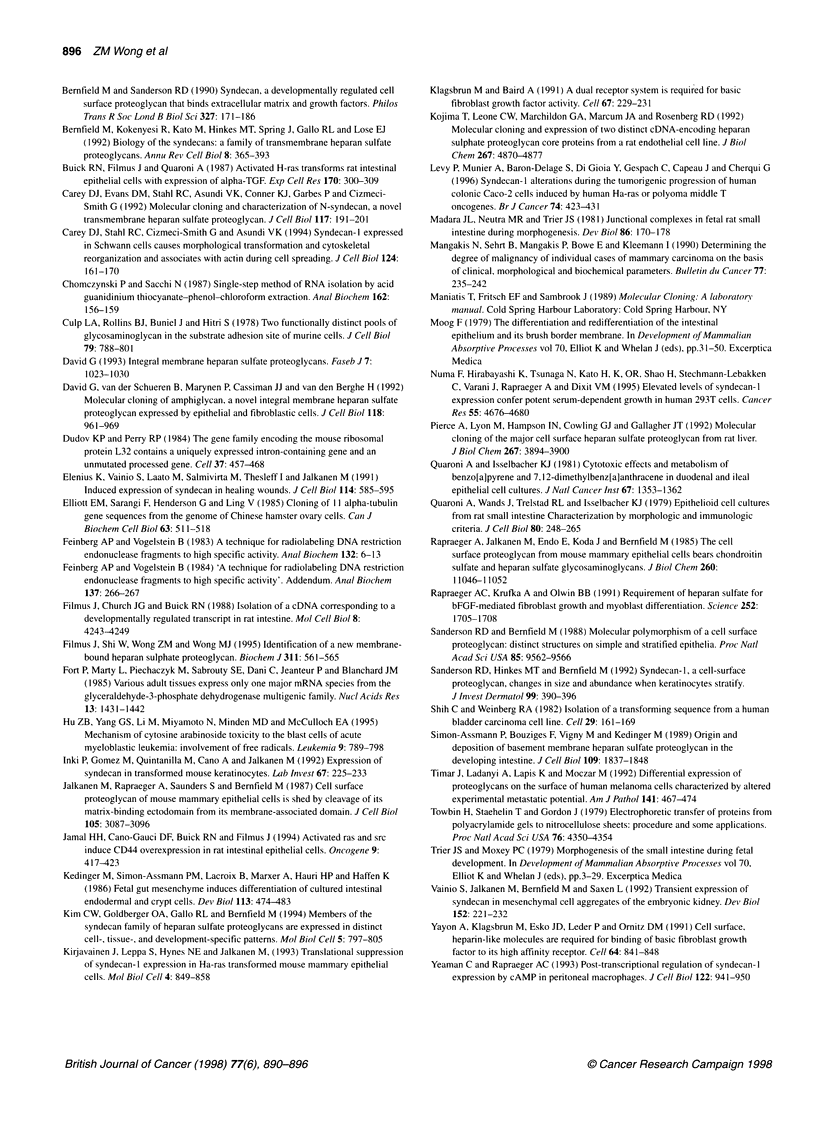


## References

[OCR_00695] Aviezer D., Levy E., Safran M., Svahn C., Buddecke E., Schmidt A., David G., Vlodavsky I., Yayon A. (1994). Differential structural requirements of heparin and heparan sulfate proteoglycans that promote binding of basic fibroblast growth factor to its receptor.. J Biol Chem.

[OCR_00710] Bernfield M., Kokenyesi R., Kato M., Hinkes M. T., Spring J., Gallo R. L., Lose E. J. (1992). Biology of the syndecans: a family of transmembrane heparan sulfate proteoglycans.. Annu Rev Cell Biol.

[OCR_00705] Bernfield M., Sanderson R. D. (1990). Syndecan, a developmentally regulated cell surface proteoglycan that binds extracellular matrix and growth factors.. Philos Trans R Soc Lond B Biol Sci.

[OCR_00715] Buick R. N., Filmus J., Quaroni A. (1987). Activated H-ras transforms rat intestinal epithelial cells with expression of alpha-TGF.. Exp Cell Res.

[OCR_00721] Carey D. J., Evans D. M., Stahl R. C., Asundi V. K., Conner K. J., Garbes P., Cizmeci-Smith G. (1992). Molecular cloning and characterization of N-syndecan, a novel transmembrane heparan sulfate proteoglycan.. J Cell Biol.

[OCR_00724] Carey D. J., Stahl R. C., Cizmeci-Smith G., Asundi V. K. (1994). Syndecan-1 expressed in Schwann cells causes morphological transformation and cytoskeletal reorganization and associates with actin during cell spreading.. J Cell Biol.

[OCR_00731] Chomczynski P., Sacchi N. (1987). Single-step method of RNA isolation by acid guanidinium thiocyanate-phenol-chloroform extraction.. Anal Biochem.

[OCR_00736] Culp L. A., Rollins B. J., Buniel J., Hitri S. (1978). Two functionally distinct pools of glycosaminoglycan in the substrate adhesion site of murine cells.. J Cell Biol.

[OCR_00741] David G. (1993). Integral membrane heparan sulfate proteoglycans.. FASEB J.

[OCR_00745] David G., van der Schueren B., Marynen P., Cassiman J. J., van den Berghe H. (1992). Molecular cloning of amphiglycan, a novel integral membrane heparan sulfate proteoglycan expressed by epithelial and fibroblastic cells.. J Cell Biol.

[OCR_00751] Dudov K. P., Perry R. P. (1984). The gene family encoding the mouse ribosomal protein L32 contains a uniquely expressed intron-containing gene and an unmutated processed gene.. Cell.

[OCR_00759] Elliott E. M., Sarangi F., Henderson G., Ling V. (1985). Cloning of 11 alpha-tubulin gene sequences from the genome of Chinese hamster ovary cells.. Can J Biochem Cell Biol.

[OCR_00764] Feinberg A. P., Vogelstein B. (1983). A technique for radiolabeling DNA restriction endonuclease fragments to high specific activity.. Anal Biochem.

[OCR_00773] Filmus J., Church J. G., Buick R. N. (1988). Isolation of a cDNA corresponding to a developmentally regulated transcript in rat intestine.. Mol Cell Biol.

[OCR_00778] Filmus J., Shi W., Wong Z. M., Wong M. J. (1995). Identification of a new membrane-bound heparan sulphate proteoglycan.. Biochem J.

[OCR_00782] Fort P., Marty L., Piechaczyk M., el Sabrouty S., Dani C., Jeanteur P., Blanchard J. M. (1985). Various rat adult tissues express only one major mRNA species from the glyceraldehyde-3-phosphate-dehydrogenase multigenic family.. Nucleic Acids Res.

[OCR_00789] Hu Z. B., Yang G. S., Li M., Miyamoto N., Minden M. D., McCulloch E. A. (1995). Mechanism of cytosine arabinoside toxicity to the blast cells of acute myeloblastic leukemia: involvement of free radicals.. Leukemia.

[OCR_00794] Inki P., Gomez M., Quintanilla M., Cano A., Jalkanen M. (1992). Expression of syndecan in transformed mouse keratinocytes.. Lab Invest.

[OCR_00797] Jalkanen M., Rapraeger A., Saunders S., Bernfield M. (1987). Cell surface proteoglycan of mouse mammary epithelial cells is shed by cleavage of its matrix-binding ectodomain from its membrane-associated domain.. J Cell Biol.

[OCR_00804] Jamal H. H., Cano-Gauci D. F., Buick R. N., Filmus J. (1994). Activated ras and src induce CD44 overexpression in rat intestinal epithelial cells.. Oncogene.

[OCR_00809] Kedinger M., Simon-Assmann P. M., Lacroix B., Marxer A., Hauri H. P., Haffen K. (1986). Fetal gut mesenchyme induces differentiation of cultured intestinal endodermal and crypt cells.. Dev Biol.

[OCR_00814] Kim C. W., Goldberger O. A., Gallo R. L., Bernfield M. (1994). Members of the syndecan family of heparan sulfate proteoglycans are expressed in distinct cell-, tissue-, and development-specific patterns.. Mol Biol Cell.

[OCR_00819] Kirjavainen J., Leppä S., Hynes N. E., Jalkanen M. (1993). Translational suppression of syndecan-1 expression in Ha-ras transformed mouse mammary epithelial cells.. Mol Biol Cell.

[OCR_00824] Klagsbrun M., Baird A. (1991). A dual receptor system is required for basic fibroblast growth factor activity.. Cell.

[OCR_00828] Kojima T., Shworak N. W., Rosenberg R. D. (1992). Molecular cloning and expression of two distinct cDNA-encoding heparan sulfate proteoglycan core proteins from a rat endothelial cell line.. J Biol Chem.

[OCR_00834] Levy P., Munier A., Baron-Delage S., Di Gioia Y., Gespach C., Capeau J., Cherqui G. (1996). Syndecan-1 alterations during the tumorigenic progression of human colonic Caco-2 cells induced by human Ha-ras or polyoma middle T oncogenes.. Br J Cancer.

[OCR_00842] Madara J. L., Neutra M. R., Trier J. S. (1981). Junctional complexes in fetal rat small intestine during morphogenesis.. Dev Biol.

[OCR_00844] Mangakis N., Sehrt B., Mangakis P., Böwe E., Kleemann I. (1990). Determining the degree of malignancy of individual cases of mammary carcinoma on the basis of clinical, morphological and biochemical parameters.. Bull Cancer.

[OCR_00860] Numa F., Hirabayashi K., Tsunaga N., Kato H., O'Rourke K., Shao H., Stechmann-Lebakken C., Varani J., Rapraeger A., Dixit V. M. (1995). Elevated levels of syndecan-1 expression confer potent serum-dependent growth in human 293T cells.. Cancer Res.

[OCR_00867] Pierce A., Lyon M., Hampson I. N., Cowling G. J., Gallagher J. T. (1992). Molecular cloning of the major cell surface heparan sulfate proteoglycan from rat liver.. J Biol Chem.

[OCR_00872] Quaroni A., Isselbacher K. J. (1981). Cytotoxic effects and metabolism of benzo[a]pyrene and 7,12-dimethylbenz[a]anthracene in duodenal and ileal epithelial cell cultures.. J Natl Cancer Inst.

[OCR_00877] Quaroni A., Wands J., Trelstad R. L., Isselbacher K. J. (1979). Epithelioid cell cultures from rat small intestine. Characterization by morphologic and immunologic criteria.. J Cell Biol.

[OCR_00888] Rapraeger A. C., Krufka A., Olwin B. B. (1991). Requirement of heparan sulfate for bFGF-mediated fibroblast growth and myoblast differentiation.. Science.

[OCR_00882] Rapraeger A., Jalkanen M., Endo E., Koda J., Bernfield M. (1985). The cell surface proteoglycan from mouse mammary epithelial cells bears chondroitin sulfate and heparan sulfate glycosaminoglycans.. J Biol Chem.

[OCR_00893] Sanderson R. D., Bernfield M. (1988). Molecular polymorphism of a cell surface proteoglycan: distinct structures on simple and stratified epithelia.. Proc Natl Acad Sci U S A.

[OCR_00898] Sanderson R. D., Hinkes M. T., Bernfield M. (1992). Syndecan-1, a cell-surface proteoglycan, changes in size and abundance when keratinocytes stratify.. J Invest Dermatol.

[OCR_00903] Shih C., Weinberg R. A. (1982). Isolation of a transforming sequence from a human bladder carcinoma cell line.. Cell.

[OCR_00907] Simon-Assmann P., Bouziges F., Vigny M., Kedinger M. (1989). Origin and deposition of basement membrane heparan sulfate proteoglycan in the developing intestine.. J Cell Biol.

[OCR_00912] Timar J., Ladanyi A., Lapis K., Moczar M. (1992). Differential expression of proteoglycans on the surface of human melanoma cells characterized by altered experimental metastatic potential.. Am J Pathol.

[OCR_00917] Towbin H., Staehelin T., Gordon J. (1979). Electrophoretic transfer of proteins from polyacrylamide gels to nitrocellulose sheets: procedure and some applications.. Proc Natl Acad Sci U S A.

[OCR_00927] Vainio S., Jalkanen M., Bernfield M., Saxén L. (1992). Transient expression of syndecan in mesenchymal cell aggregates of the embryonic kidney.. Dev Biol.

[OCR_00932] Yayon A., Klagsbrun M., Esko J. D., Leder P., Ornitz D. M. (1991). Cell surface, heparin-like molecules are required for binding of basic fibroblast growth factor to its high affinity receptor.. Cell.

[OCR_00937] Yeaman C., Rapraeger A. C. (1993). Post-transcriptional regulation of syndecan-1 expression by cAMP in peritoneal macrophages.. J Cell Biol.

